# Vulvar Pruritus: A Review of Clinical Associations, Pathophysiology and Therapeutic Management

**DOI:** 10.3389/fmed.2021.649402

**Published:** 2021-04-07

**Authors:** Haya S. Raef, Sarina B. Elmariah

**Affiliations:** ^1^Tufts University School of Medicine, Boston, MA, United States; ^2^Massachusetts General Hospital, Boston, MA, United States

**Keywords:** vulva, pruritus, itch, neurogenic itch, barrier function, neuropathic itch

## Abstract

Vulvar pruritus is an unpleasant sensation and frequent symptom associated with many dermatologic conditions, including infectious, inflammatory and neoplastic dermatoses affecting the female genitalia. It can lead to serious impairment of quality of life, impacting sexual function, relationships, sleep and self-esteem. In this review, common conditions associated with vulvar itch are discussed including atopic and contact dermatitis, lichen sclerosus, psoriasis and infectious vulvovaginitis. We review the potential physiologic, environmental and infectious factors that contribute to the development of vulvar itch and emphasize the importance of addressing their complex interplay when managing this disruptive and challenging symptom.

## Introduction

Vulvar pruritus is a frequently chronic and debilitating symptom associated with many vulvar disorders. The exact prevalence of vulvar pruritus is unknown as epidemiologic data are limited and most reports focus on individual conditions involving genital itch. In a study that surveyed 480 women from the general population in Boston, Massachusetts, 6.6% of women reported experiencing vulvar itch or burning (1). This is similar to rates of vulvar pruritus reported amongst the general German population, which was noted to range from 5 to 10% (2). The true prevalence of vulvar pruritus may be difficult to assess as it is likely underreported given the embarrassment many women feel when discussing genital symptoms (3). Itch was found to be the most frequent symptom, occurring in 70% of patients presenting to a clinic specializing in vulvar conditions (4). Similarly, in a survey study performed in the United Kingdom, 67.3% of general practitioners reported that they see more than five patients per month with vulvar symptoms, with vulvar pruritus being the most common (5).

Vulvar pruritus can have a profound impact on quality of life (6). In patients with genital psoriasis, itch was reported to be the most bothersome symptom with substantial impact on sexual relationships and psychosocial well-being (7). Moreover, several studies have demonstrated the negative impact of lichen sclerosus, a condition characterized by genital itch and pain, on sexual satisfaction (8–10).

## Pruritic Vulvar Dermatoses

Vulvar pruritus arises in the setting of many inflammatory, infectious, and neoplastic processes that can affect the female genitalia (11).

### Inflammatory

#### Common Etiologies

Common inflammatory vulvar dermatoses characterized by marked pruritus include atopic and contact dermatitis, lichen planus, lichen simplex chronicus, psoriasis and lichen sclerosus, among others. Atopic dermatitis (AD), irritant contact dermatitis (ICD) and allergic contact dermatitis (ACD) are the most common causes of vulvar itch in women (12). In one study of 144 adult women with vulvar complaints, 66% of patients had an eczematous process confirmed on pathology (13). In a separate prospective cohort study, 81.4% of patients with vulvar itch were found to have at least one positive contact allergen on patch testing (14). Similarly, AD and ICD have been reported to be the most common cause of vulvar symptoms in prepubertal girls (15).

AD is a genetic skin disease characterized by an altered skin barrier and chronic pruritus. It presents acutely as erythematous edematous or vesiculated plaques. Lichenification and dyspigmentation may be observed in chronic cases. It is important to note that due to the altered skin barrier, patients with AD may be at higher risk for the development of both irritant and allergic contact dermatoses (16–19).

Contact dermatitis consists of inflammation of the skin resulting from an external agent that acts as an irritant or as an allergen. The manifestation of both forms of dermatitis is very similar, varying from mild erythema and scaling to more severe erythema and edema (20). The area of involvement may be localized to the exposed site or be more extensive as the product spreads, with moisture or scratching (20). In addition to itch, pain and burning may also be present. Ulceration and erosions may be seen with primary irritants (21). In ACD, vesiculation in the acute phase may be observed (22). In more chronic cases, lichenification with excoriation are common features. Secondary infection can be seen in both ICD and ACD with pustules, crusting and fissuring.

Many substances can cause irritation of the vulva, including body fluids, feminine hygiene products or various topical medications (20). Physical and thermal irritants like tight fitting clothes, wash cloths, sponges and hair dryers have been implicated in ICD development (20, 22). Similarly, allergens often contribute to itch and dermatitis in patients with vulvar disease. Common allergens include fragrances and preservatives in products like soaps and detergents, cleansing wipes, antiseptics, spermicides, sanitary pads, lubricants, and even topical treatments like steroids, anesthetics, antibacterial and antifungal agents (20, 23). Rubber products, like pessaries, condoms, diaphragms, and gloves may also be sensitizers (20). Additionally, pantyhose and clothing with azo dyes may contribute to ACD (20). Patch testing may identify the allergens responsible for ACD.

Lichen simplex chronicus (LSC), or circumscribed neurodermatitis, is an eczematous disorder that commonly affects vulvar skin. It presents as scaly, thickened plaques that develop in response to persistent and vigorous scratching of intensely pruritic sites (24). LSC accounts for 35% of patient visit to vulvar specialty clinics, predominately affecting adult women but may also occur in children (25). Although often considered a primary diagnosis, LSC often arises as a secondary finding in the setting of neuropathic or other underlying primary cutaneous diseases such as AD, ACD or LP (26). It can also occur in patients with psychiatric disorders like depression and obsessive-compulsive disorder (27, 28). Pruritus in systemic diseases such as end stage renal disease, obstructive biliary disease or Hodgkin's lymphoma can also provoke LSC (29). LSC is characterized by a self-perpetuating itch-scratch cycle. In patients with primary LSC, the itch-scratch cycle is often triggered by initial skin irritation from tight-fitting clothing, irritating fabrics or personal care items which provoke scratching (11, 30). Damage to the vulvar epithelium due to scratching compromises the skin barrier, potentially provoking release of epithelial cytokines and making the skin more vulnerable to potential infection, which in turn drives itch and inflammation (26). If sufficiently pronounced, scratching results in hypertrophy and lichenification of the genital skin. Clinical examination usually reveals lichenified plaques with excoriations and variable levels of erythema and scale (25).

Psoriasis is another common inflammatory skin disease that affects genital skin and is often accompanied by pruritus (31). In most cases, genital psoriasis arises in the setting of more widespread cutaneous involvement, but isolated presentation of genital psoriasis may occur in 2–5% of psoriatic patients (32). Psoriatic lesions of the vulva are more common in children than in adults. In a study that evaluated 130 prepubertal girls with vulvar complaints, 17% had psoriasis, which was the third most common cutaneous condition after AD and LS (15). Clinical features of vulvar psoriasis consist of well-demarcated, brightly erythematous plaques with or without scale on the labia majora (33). Plaques may extend to the inguinal folds and maceration may be present (27).

Lichen sclerosus (LS) is another inflammatory dermatosis that affects the vulvar and vaginal mucosa, and not uncommonly extends to the perineum and perianal skin. While vulvar LS can occur at any age, most cases are observed in prepubertal girls or in postmenopausal women, when endogenous estrogen production is low (34). Pruritus and pain are predominant symptoms of the disease, although rarely LS may be asymptomatic. Other symptoms include dyspareunia and dysuria. In children, constipation may be a presenting symptom due to pain with defecation (35). The exact prevalence of LS is unknown, but estimates range from 1:300 to 1:1,000 of all adult patients referred to dermatology departments (36). The estimated prevalence in prepubertal girls is 1 in 900 (37). At a general gynecology practice, the rate of vulvar LS was estimated to be 1.7% (38). Again, the discrepancy in reported prevalence among gynecology and dermatology practices may reflect the hesitance of patients to discuss genital symptoms outside of a dedicated health visit focused on genitourinary care. LS typically manifests as atrophic, pale to white patches or plaques that often form a figure-of-eight pattern encompassing the vulva and anus (39). Erosions and painful fissures may be observed due to underlying inflammation as well as scratching or irritation of the inflamed, fragile tissue. Loss of normal vulvar architecture may be observed in more advanced cases, with burying of the clitoris and agglutination of the labia. Lichen sclerosus is associated with increased risk of developing genital squamous cell carcinoma (SCC). While the exact risk of malignant transformation is uncertain, estimates of the development of SCC are between 3 and 5% (40, 41). In a more recent study that used data from the Dutch Pathology Registry and included 3,038 women diagnosed with lichen sclerosus, the risk of SCC development after 10 and 20 years was 3.3 and 6.7%, respectively (42).

Lichen planus (LP) is a highy pruritic, autoimmune mucocutaneous disorder in which activated T-cells target basal keratinocytes of keratinized and non-keratinized squamous epithelium (43). The prevalence of LP is estimated to be 1% of the general population (44). Although LP most commonly affects the oral mucosa, ~25% of women with oral LP also have vulvovaginal involvement (45). LP predominately affects adult women, although isolated cases have been reported in young girls (46). Vulvovaginal LP may manifest in several ways, presenting in erosive, papulosquamous, and hypertrophic forms (47). Erosive LP, the most common presentation affecting genital skin, is characterized by well-demarcated glassy, erythematous to violaceous patches with a hyperkeratotic border and primarily affects the non-keratinized epithelium of the vestibule, labia minora and vagina (48). Several cohort studies have examined the distribution of LP subtypes arising on keratinized skin of the labia majora (48, 49). Papulosquamous LP, also referred to as classic LP, arises in 4–33% of cases and manifests with purple, brown or pink polygonal papules or small plaques which may have associated Wickham's striae (48, 49). Hypertrophic lichen planus arises in 29–46% of cases and presents with thicker, violaceous and hyperkeratotic plaques (47). One cohort study describing clinical and histologic features in 63 vulvar LP cases reported pruritus as the primary symptom affecting 79 and 81% of hypertrophic and papulosquamous LP patients, respectively, while pain was a primary manifestation in only 10 and 14%, respectively (48). Similar to LS, longstanding and untreated disease may lead to alterations in the normal architecture with narrowing of the vaginal introitus (50).

#### Other Etiologies

Inflammatory vulvar pruritus may also be caused by seborrheic dermatitis, plasma cell vulvitis, and Fox-Fordyce disease. Seborrheic dermatitis is an inflammatory condition that affects the sebum-rich areas of the body and should be considered in patients with vulvar pruritus. While uncommon, seborrheic dermatitis can occasionally present on the vulva, and is usually associated with simultaneous appearance of characteristic seborrhea on the scalp and face (51). It manifests on the vulva as erythematous plaques mainly on the labia, majora, perineum, and mons pubis. Scale is frequently absent in the vulva and the severity of pruritus is often more marked than would be expected based on the clinical signs (52).

Plasma cell vulvitis (PCV) is an extremely rare cause of vulvar pruritus characterized by a well-circumscribed erythematous plaque composed of predominately plasma cells (53, 54). It is typically located within the vulvar vestibule, often extending to the medial labia minora. The most common symptoms associated with PCV are pruritus, pain, burning, and dyspareunia (55).

Fox-Fordyce disease is another rare inflammatory condition which can affect vulvar skin and may provoke intense itching. The primary pathophysiologic process involves obstruction of the apocrine sweat duct and subsequent ductal rupture causing inflammation and enlargement of the glands (56). The mons pubis and labia majora are most commonly affected. Clinically, Fox-Fordyce disease manifests as intensely pruritic, numerous, flesh-colored to slightly yellow papules (57, 58).

### Infectious

#### Common Etiologies

Vulvar pruritus may be associated with several types of infections and these vary with age. In prepubertal females, infection with *Group A beta-hemolytic streptococcus* (GABHS) commonly provokes vulvar symptoms including pruritus and pain, and manifests with sharply demarcated, edematous, red plaques (59). In contrast, adult women are less susceptible to acute GABHS-mediated vulvitis. Oropharyngeal GABHS infection often, but not always, precedes the development of vulvar symptoms (60).

In adult women, vulvovaginal candidiasis is a frequent cause of vulvar pruritus, with some studies suggesting candidiasis accounts for 35–40% of vulvar itch cases in this age group (2). Multiple epidemiologic studies have indicated that *Candida albicans* is responsible for the excess of episodes of vulvovaginal candidiasis, although reports indicate than non-*albicans Candida* species, notably *Candida glabrata*, account for 10–20% of episodes in certain regions (61–63). Increased estrogen levels, which have been implicated in reducing the inhibitory activity of epithelial cells against *Candida*, are thought to account for the rise in candidiasis in women of reproductive age (64). It is estimated that 75% of women have been affected by vulvovaginal candidiasis at some point in their lifetime (61, 65). Pregnancy, antibiotics, oral contraceptives and hormonal replacement therapies may increase estrogen levels resulting in an increased frequency of disease (66, 67). Tamoxifen, an estrogen antagonist in breast tissue, has been reported to have estrogen-like effects on vaginal epithelium in postmenopausal women, increasing risk of vulvovaginal candidiasis (68–70). In addition, compromised immune function is also associated with increased risk of yeast infections, as has been observed in patients with diabetes, HIV or who regularly use systemic or topical corticosteroids (23). Patients with recurrent candidal vulvovaginitis, defined as the occurrence of at least four episodes in 1 year, may have a predisposing genetic factor underlying their susceptibility (71). Clinical presentation of vulvar erythema, pustules or erosions and vaginal discharge may vary, but symptoms of pruritus and burning are commonly observed. Additional symptoms may include dysuria and dyspareunia. Identification of the specific *Candida* species can be considered in patients with refractory or recurrent vulvovaginal candidiasis as some species are often resistant to treatment (72).

#### Other Etiologies

In contrast to GABHS, which commonly affects prepubertal females, *Staphylococcus aureus, Haemophilus* and *Shigella* infections are less common infectious causes of vulvovaginal pruritus (23). E*nterobius vermiuclaris* (pinworm) infestation may be another source of vulvar and perineal pruritus in younger females worldwide (73).

In adults, the two most common parasitic vulvar infestations are pediculosis pubis (pubic lice) and scabies (52). Both cutaneous infections are often seen in young adults and are typically acquired during sexual contacts. Vulvar pruritus is the predominant symptom that develops following allergic sensitization (52, 74). In pediculosis pubis, adult lice and their eggs (nits) can be visible to the naked eye. Infection may spread from the genital area to other parts of the body, such as the thighs or trunk (74). Infestation with scabies causes widespread itching with nocturnal predominance. Unlike in other areas of the body, burrows on the vulva are uncommon and may be masked by excoriations or secondary infection (52).

Tinea cruris is an additional infection that can cause vulvar pruritus in women (52, 75). It can involve the inguinal creases and the labia majora. The typical lesions consist of mildly pruritic plaques with a raised erythematous scaly edge and central clearing. Viral infections, such as herpes simplex virus (HSV), human papilloma virus (HPV), and molluscum contagiosum may also trigger a sensation of vulvar itch (52). However, herpetic infections predominately manifest as pain, and HSV and molluscum are typically asymptomatic.

### Neoplastic

Benign or malignant neoplasms are uncommon causes of vulvar pruritus. Rarely, pruritus may be an indication of vulvar malignancy such as SCC, melanoma, extramammary Paget's disease (EMPD) or vulvar intraepithelial neoplasia (VIN). Vulvar malignancy is uncommon and represents approximately 2–5% of all gynecologic cancers, with SCC representing the vast majority (>80%) of cases, followed by melanoma, BCC, verrucous carcinoma, EMPD, adenocarcinoma and Bartholin gland carcinoma (76). Although frequently overlooked, pruritus is the most common initial symptom of vulvar malignancy, with reports of up to 50–60% of patients endorsing moderate to severe pruritus (11, 77). In a multi-center, retrospective study describing 76 women with vulvar cancer in Tunisia, 48.7% of patients experienced chronic pruritus as the presenting symptom and the mean interval of time from symptom onset to cancer diagnosis was ~12.9 months (+/– 6.38) (77). Squamous cell carcinoma typically presents as persistent papules, plaques or ulcers with associated bleeding, itch and/or pain that is refractory to anti-inflammatory treatment (78). It is more common in postmenopausal women and is often associated with LS. Paget's disease of the vulva is an uncommon lesion that represents <1% of vulvar neoplasms (79). It predominately affects postmenopausal Caucasian women and presents as a white to red, velvety pruritic thin plaques (80). Although usually confined to the epithelium, invasive disease is observed in 15–25% of patients (81). VIN is a premalignant finding and is associated with HPV infection, particularly subtypes 16 and 18 (82). It can cause itch leading to varying degrees of excoriation and crusting (11).

Additionally, a variety of benign neoplastic processes may contribute to vulvar pruritus. For example, syringomas are rare tumors derived from eccrine sweat glands. While they typically involve the face, neck or chest, they occasionally present as multiple small, flesh-colored pruritic papules on the vulva (83). Moreover, hidradenoma papilliferum (HP), a tumor thought to originate from apocrine glands or mammary glands, can occasionally occur on the vulva and cause pruritus (84). In one series, HP represented up to 60% of vulvar adnexal tumors (85). It usually manifests as a firm, flesh to red-colored nodule that may or may not be accompanied with pruritus (86). It can be confused with adenocarcinoma due to its tendency to ulcerate (83, 87).

## Pathophysiology of Vulvar Itch

### Impaired Barrier Function

Many pruritic vulvar disorders, such as atopic dermatitis and psoriasis, are associated with altered skin barrier function (88). Disruption of the skin barrier can be caused from a variety of factors including epidermal inflammation and mechanical or environmental insults, which in turn activate itch receptors (89). The barrier function of vulvar skin is substantially weaker than at other anatomical sites, and thus may be particularly prone to developing pruritus. The rate of transepidermal water loss (TEWL), a marker of barrier function, is significantly higher in vulvar skin than the skin of other cutaneous sites such as the forearm, suggesting a weaker epidermal barrier at the vulva (90, 91). Indeed, several studies have shown vulvar skin to be more reactive to irritants compared to other skin areas. In one study, two irritants, bezalkonium chloride and maleic acid, were applied the labia majora and forearm, and the intensity of skin reactions were assessed (92). Vulvar skin was found to be significantly more reactive than forearm skin to the two irritants, although this reactivity was not reproduced in studies with another irritant, sodium lauryl sulfate (93). Sweat, urine, friction by clothes and feminine hygiene products may all contribute to vulvar irritation by weakening barrier function (94). Moreover, low estrogen levels occurring with menopause, breast-feeding, postpartum and medications can also result in impaired barrier function as estrogen is important to maintain the structural integrity of the vulvovaginal space (20). Thinning of the vulvar epithelium in postmenopausal women combined with elevated skin pH and reduced corneum hydration cause barrier dysfunction (95).

Once disrupted, the skin barrier is more susceptible to exogenous and endogenous itch-triggers. In addition to the potential itch or pain associated with microbial colonization, mechanical irritation and chemical injury discussed above, epithelial damage leads to immune activation via release of skin-specific cytokines, including thymic stromal lymphopoietin (TSLP) and interleukin (IL)-33, which directly activate pruriceptive afferent nerve fibers (96, 97). Moreover, cysteine and serine proteases, such as cathepsin S and various kallikreins (KLKs), may be released by keratinocytes upon barrier disruption and are capable of directly stimulating or modulating itch via activation of Mas-related G-protein coupled receptors (MRGPRs) and protease-activated receptors (PARs) (98–100).

### Neural Dysfunction

Neural dysfunction, due to neurogenic or neuropathic insults, is common but often-overlooked cause of vulvar pruritus. Neurogenic itch originates from endogenous or exogenous factors that activate the central nervous system at the level of the brain or spinal cord without evidence of nerve damage (101). Growing evidence suggests that neurogenic factors may contribute to vulvar pruritus. Epithelial and stromal tissue of the vulvar skin and vaginal mucosa express the transient receptor potential cationic channel type A1 (TRPA1), a channel known for its role in mediating and modulating non-histaminergic itch (102). Animal models of neonatal vaginal irritation suggest that hypersensitivity of the vagina is driven in part by increased hypothalamic-pituitary-adrenal (HPA) axis activation and subsequent increases in TRPA1 expression and functional activity in nerve terminals innervating the vaginal mucosa (103). Similarly, separate studies have demonstrated that expression of the transient receptor potential vanilloid 1 (TRPV1) ion channel, also well-known for its role in modulating pain and itch signals, is increased in vulvovaginal epithelia in patients with vulvodynia compared to controls (104, 105). Although vulvodynia is classically regarded as a type of neuropathic pain, itch and burning can accompany vulvodynia in 20 and 70%, respectively (106). Future studies will be needed to specifically evaluate whether the expression and function of TRP ion channels as well as primary itch-sensing receptors are altered in disorders associated with vulvar pruritus.

In contrast to neurogenic pruritus in which neural architecture is considered normal but stimulated abnormally, neuropathic pruritus results from injury or damage to nerve fibers. Small fiber polyneuropathy (SFPN), which affects the small, unmyelinated C-fibers and thinly myelinated A-delta fibers that conduct itch and pain may arise secondary to systemic diseases such as diabetes mellitus, sarcoidosis, amyloidosis, B12 deficiency, and viral infections, among others (107). While individuals with SFPN usually present with symptoms in their distal extremities or generalized symptoms, itch can also be localized entirely to the vulva. Vulvar itch may also be caused by nerve or nerve root compression at the levels of L4 through S2 vertebrae secondary to spinal injuries or lumbosacral arthritis (11, 23, 108). Another source of potential nerve irritation or injury may be caused by reactivation of varicella zoster, as 8.4% of shingles cases affect the dermatomes that innervate the vulva (109). Despite a robust immune response, long-lasting damage to the affected nerves may result in persistent pain and/or itch in affected vulvar skin (110). It is estimated that 30% of people with post-herpetic neuralgia suffer from itch (111), and thus postherpetic itch (PHI) should be considered in women presenting with genital pruritus.

### Hormonal Influence

Hormonal changes play an important role in regulating vulvar epithelium by influencing vaginal pH and microflora composition. Similar to the vagina, vulvar pH is related to hormonal status and will change over a lifetime (112). In childhood, the vulvar and vaginal epithelia are neutral or alkaline, due to a lack of acid-producing vaginal microbes, lactobacilli (113). With the onset of menstruation, cyclic changes in estrogen and progesterone create a new epithelial micro-environment. Estrogen stimulation increases glycogen levels in the vulvar epithelium and lactobacilli subsequently colonize the vulvovaginal area, causing the pH to decrease (114). During parts of the menstrual cycle and following menopause, decreases in systemic estrogen result in an increase in vulvovaginal pH.

At more alkaline pH, the activity of various epithelial or immune-cell derived proteases may increase and thereby lead to greater activation of neuronal itch receptors (100, 115). Consistent with this hypothesis, abnormal expression of proteases and PAR activation has been implicated in several pruritic inflammatory skin disorders, such as AD and psoriasis (116–119). In addition to their effects on neuronal PARs, serine proteases such as KLKs and mast cell tryptase may also activate PARs expressed by keratinocytes or endothelial cells, stimulating the release of neuropeptides and cytokines which drive neurogenic inflammation and further propagation of endogenous pruritogens (120–123). Furthermore, the interactions between proteases and their endogenous inhibitors, present in the skin to ensure skin homeostasis, are influenced by the pH, with the greatest inhibitory capacity occurring in neutral pH environments (124). Thus, fluctuations in vulvovaginal pH due to hormonal status could shift the balance between protease and protease inhibitor activity, further contributing to PAR-mediated inflammation and itch.

### Microbiome

The composition of the human vaginal microbiome may contribute to the pathogenesis of vulvar pruritus, particularly with respect to itch triggered by the mucocutaneous pathogens discussed previously. Interestingly, compared to the microbiota that colonize other regions of the body, such as the oropharynx and gut, the vaginal microbiome exhibits much lower diversity with Lactobacillus as the dominating species (125). Vaginal pH correlates with microbiome composition. Indeed, the composition of the vulvovaginal microbiome is dynamic and influenced by hormone-driven pH changes throughout the woman's reproductive life (126). Lactobacillus dominance increases with high estrogen levels because of proliferation and accumulation of glycogen. Ethnic differences also correlate with microbiome composition, with Blacks and Hispanics demonstrating higher levels of anaerobic bacterial species (125–127). By acidifying the vagina and producing antimicrobial substances, such as lactic acid and hydrogen peroxide, lactobacilli protect against opportunistic infections (2). Similarly, in keratinized squamous epithelia like that of the labia majora, resident microbiota such as *Cutibacterium acnes* promotes reduces skin pH via production of short-chain fatty acids (128). Other commensal microbes, such as *Staphylococcus epidermidis*, not only produce biofilms and other enzymes that enhance the function of antimicrobial peptides (AMPs) such as human b-defensins (HbDs) against dysbiosis, but also promote production of anti-inflammatory cytokines such as IL-10 from antigen presenting cells and reduce pro-inflammatory signals released by keratinocytes (129, 130).

Dysbiosis may lead to pruritus or sensory disturbance via multiple mechanisms. First, bacterial and viral pathogens directly engage keratinocytes via cell surface toll-like receptors (TLRs), triggering their release of the alarmins TSLP and IL-33 as well as AMPs such as HbDs and canthelicidins. While the alarmins directly activate itch by binding to their receptors on peripheral afferents (96, 97), they also initiate T_H_2 immune cascades that contribute to barrier inflammation that fuels ongoing pruritus (131). Keratinocyte-derived AMPs trigger itch indirectly by stimulating mast cell release of histamine and IL-31 which in turn activate pruriceptors (132). Moreover, mast cells may also detect microbiota via their own TLRs or their ability to respond to a host of endogenous molecules released as part of a coordinated tissue response to infection including substance P and complements (133). Consistent with these data, derived primarily from animal models and human studies in allergic disorders, one study found that compared to healthy controls, women with LS has increased expression of AMPs including HbD-2 and psoriasin (134).

Pathogenic bacteria, such as *Staphylococcus aureus, Streptococcus pneumoniae, Pseudomonas aeruginosa* and others have also been shown to directly activate peripheral afferent nociceptive fibers (135). In animal models, bacteria-derived N-formylated peptides and the pore-forming toxin a-haemolysin stimulated calcium influx in nociceptive dorsal root ganglia by binding to neuronal formyl-peptide receptor 1 or by direct pore-formation, respectively (135). Once activated, nociceptors are capable of releasing neuropeptides that in turn modulate the inflammatory response, which may further influence the development of pain and/or itch. Similarly, in colonic epithelium, bacterial cell products have also been shown to directly activate dorsal root ganglion neurons and subsequently trigger elaboration of inflammatory cytokines (136). In addition, lipopolysaccharide (LPS) contained in the cell envelope of Gram-negative bacteria is also capable of stimulating calcium influx in trigeminal dorsal root ganglia neurons and sensitizing TRPV1 via a TLR4-mediated mechanism (137). How these processes specifically contribute to the development of itch or sensory disturbance in vulvovaginal epithelia remains to be examined.

## Management Considerations

Recognizing the numerous factors that contribute to the pathogenesis of vulvar pruritus is crucial for appropriate diagnostic evaluation and management. The approach to therapy should be directed against the primary underlying mechanism suspected (e.g., inflammation due to AD or ACD, candidiasis, etc), but must also account for other exacerbating factors.

### Pharmacologic Treatments

Topical corticosteroids are commonly used to alleviate itch caused by inflammatory skin disease (26). The potency used in a patient should be determined based on the age of the patient, diagnosis and severity of symptoms. Some vulvar conditions, such as LS and LP, may require more potent or prolonged corticosteroid therapy like clobetasol and halobetasol, whereas a less potent formulation may be sufficient for other diagnoses (52). The topical calcineurin inhibitors, tacrolimus or pimecrolimus, are non-steroidal anti-inflammatory agents that may also be useful in reducing vulvar inflammation or pruritus, particularly when prolonged courses are required to avoid steroid-induced side effects. Additional topical therapy targeting pruritus may be considered, for instance capsaicin or doxepin preparations (101). Doxepin should be used with caution due to high sensitizing capacity (138, 139). If capsaicin cream is being considered for use on genital skin, lower concentrations (0.012%) are advised (140). Systemic steroid preparations should be considered only after topical approaches have been exhausted or in severe dermatitis. Systemic immunomodulators, such as azathioprine, methotrexate, mycophenolate mofetil and infliximab have been evaluated for their potential use in AD, however further research is needed to specifically determine their utility in pruritic vulvar dermatoses (141–144).

Excoriated epidermis can become superinfected, and thus practitioners should have a low threshold to investigate and treat potential fungal and/or bacterial superinfections even when women have other underlying reasons for genital pruritus. For infectious causes of vulvar pruritus, treatment should depend on the inciting pathogen. Specific single bacterial infections require appropriate topical or oral antibiotics. For fungal infections like candidiasis, topical or oral azole agents are effective. There is no evidence to suggest that a specific azole results in better cure rates (72, 145). However, it is important to note that *Candida glabrata* is less responsive to azoles. Vaginal boric acid or amphotericin B can be used for refractory cases (72). Treatment of pediculosis and scabies infestations is best accomplished with permethrin or pyrethrins with piperonyl butoxide (146). Accepted therapies for pinworms include pyrantel pamoate or mebendazole, which should be administered to all household members (147). For viral causes of vulvar pruritus, acyclovir or valacyclovir are considered standard treatment for genital herpes (147). In patients with condyloma acuminatum caused by HPV infection, treatment with podophyllin, liquid nitrogen, or imiquimod are effective (147).

Although few studies address using neuromodulators for neuropathic vulvar itch, some reports suggest that oral gabapentin and topical lidocaine may be effective (148, 149). Use of tricyclic antidepressants, selective serotonin reuptake inhibitors and other antidepressants may also be considered and have shown benefit in patients with chronic itch and prurigo (5). In postmenopausal women or in the setting of a hypoestrogenic state, topical estrogen therapy may be suitable to reduce symptoms of dryness, atrophy and pruritus. As our understanding of itch pathophysiology grows, targeted anti-pruritic treatments may emerge and will need to be evaluated in randomized control trials for vulvar pruritus.

### Non-pharmacologic Treatments

Because barrier dysfunction arises so frequently in conditions associated with vulvar pruritus, it is important to counsel patients to avoid all sources of irritation or potential allergic sensitization, including fragrances, lubricants and cleaning products. Patch testing should be performed for patients with physical exam or histologic findings suspicious of allergic contact dermatitis. Patch testing may also be a useful tool in patients with persistent vulvar symptoms that is unresponsive to treatment after 8 weeks, to avoid delays in diagnosis (150). Simple steps of cleansing with mild or no detergents when bathing and rinsing genital skin with water following urination when possible should be emphasized. Moreover, lipid-replenishing formulations such as petrolatum or barrier creams such as zinc oxide paste should be used to enhance barrier function of the vulvar skin and mucosa.

Behavioral modification strategies, such as skin rubbing and cooling rather than scratching can be effective when used in combination with pharmacotherapy. Patients should also be advised to keep fingernails short to minimize trauma caused by excoriation. Moreover, psychological interventions to control the urge to scratch, such as cognitive behavioral therapy, may be beneficial for some patients. A randomized controlled trial with AD patients receiving cognitive-behavioral treatment have shown significantly decreased itch intensity and scratching behavior after 1 year, as compared to those receiving only standard dermatologic care (151).

Phototherapy is another therapeutic modality that may be considered for the management of itch in various inflammatory pruritic conditions affecting the vulva, such as AD, psoriasis and LS (152). Several studies have documented the efficacy of phototherapy at various wavelengths for improving AD severity and associated pruritus, with medium-dose ultraviolet A (UVA) and narrowband UVB (NBUVB) being the preferred modalities (152–155). Similarly ultraviolet B (UVB) has been shown to reduce itch in psoriasis patients (156). Phototherapy on genital skin may best be considered in refractory cases and when handheld devices are available.

## Conclusions

Vulvar pruritus is a common symptom of multifactorial etiology that may be driven by primary inflammatory disorders, barrier disruption, hormonal changes and infectious causes. Vulvar itch has a significant impact on the quality of life of affected patients and should be addressed by gynecologists, dermatologists, urologists and general practitioners when possible. Effective therapeutic strategies require that practitioners understand the multidimensional nature of vulvar pruritus and simultaneously address the many contributing factors that underly this challenging symptom.

## Author Contributions

HR conducted the literature review and drafted the manuscript. SE participated in reviewing and revising the manuscript. Both authors read and approved the final version of the manuscript including references.

## Conflict of Interest

The authors declare that the research was conducted in the absence of any commercial or financial relationships that could be construed as a potential conflict of interest.
